# Veno-Venous Extracorporeal Membrane Oxygenation (VV-ECMO) as Treatment for Transfusion-Related Acute Lung Injury

**DOI:** 10.7759/cureus.93347

**Published:** 2025-09-27

**Authors:** Steven Douedi, Ashraf Sliem, Mihir Odak, Muhammad Raza, Jonathan Stoll, Joseph Lee

**Affiliations:** 1 Cardiology, Deborah Heart and Lung Center, Browns Mills, USA; 2 Internal Medicine, Hackensack Meridian Health (HMH) Southern Ocean Medical Center, Manahawkin, USA; 3 Critical Care, Deborah Heart and Lung Center, Browns Mills, USA

**Keywords:** blood transfusion reaction, respiratory failure, taco, transfusion related acute lung injury, vv ecmo

## Abstract

Transfusion-related acute lung injury (TRALI) manifests as acute lung injury and hypoxemia occurring several hours after transfusion. While treatment typically involves cessation of the transfusion and supportive measures, severe cases may necessitate mechanical ventilation, including the initiation of veno-venous extracorporeal membrane oxygenation (VV-ECMO). In this case, we present a severe instance of TRALI after a blood transfusion during a femoral endarterectomy. The patient's condition deteriorated intra-operatively post-transfusion, marked by worsening hypoxia and ventilator dyssynchrony, necessitating increased sedation, paralytics, and inhaled nitric oxide. With the patient’s respiratory status continuing to decline, VV-ECMO was initiated, resulting in complete recovery of lung function. Our report underscores the rare recovery of TRALI with ECMO, highlighting its efficacy during the acute phase of a transfusion reaction.

## Introduction

Transfusion reactions are adverse events that may occur following the administration of blood products, including whole blood or its components. Among these, pulmonary transfusion reactions can result in particularly severe outcomes. Transfusion-related acute lung injury (TRALI) is a clinical syndrome resembling acute respiratory distress syndrome (ARDS), characterized by the rapid onset of hypoxemia and noncardiogenic pulmonary edema within hours of transfusion [1,2]. According to the U.S. Food and Drug Administration, TRALI remains the second leading cause of transfusion-related mortality, following transfusion-associated circulatory overload (TACO) [2]. This case report describes the utilization of veno-venous extracorporeal membrane oxygenation (VV-ECMO) as a life-saving intervention for TRALI.

## Case presentation

**Figure 1:**
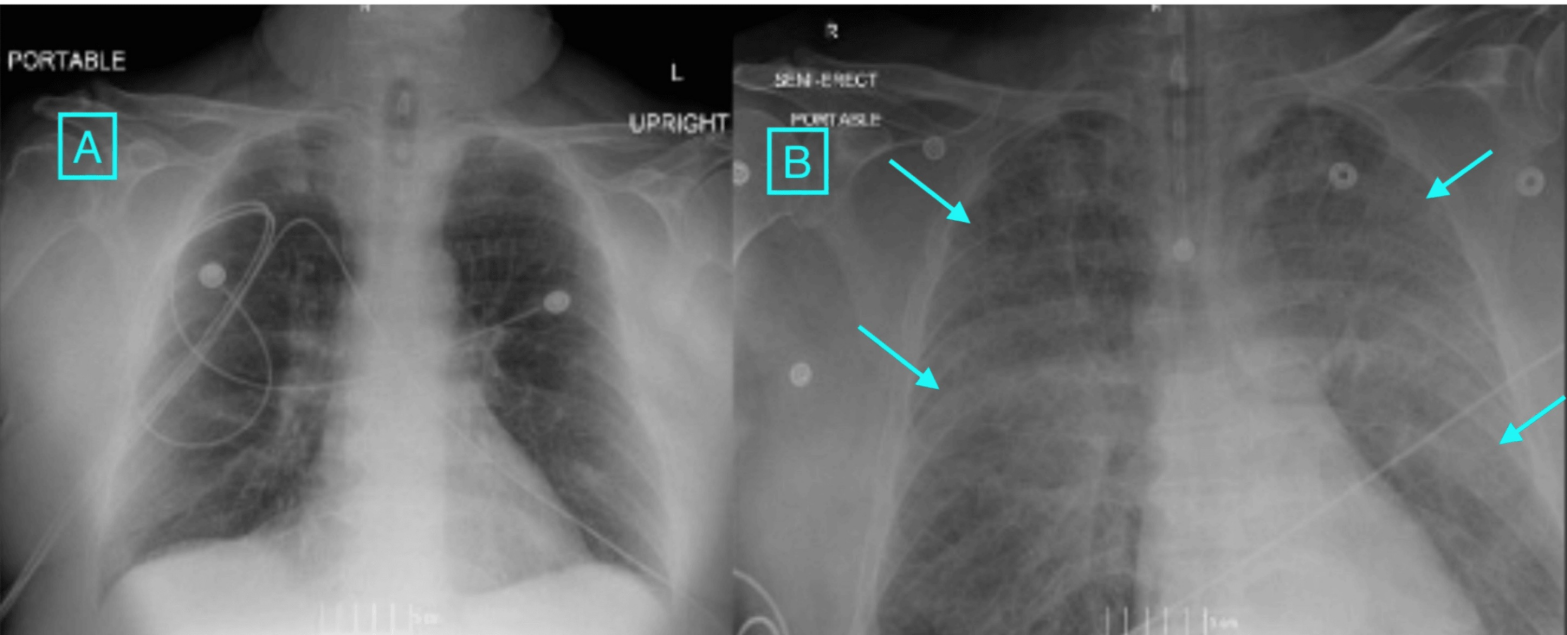
Chest X-ray showing the development of bilateral infiltrates (arrows) post-transfusion (B) when compared to pre-operative (A) with clear lungs

A 62-year-old male with a history of coronary artery disease, chronic kidney disease, hypertension, hyperlipidemia, 40-pack-year tobacco use, and type 2 diabetes mellitus who initially presented to our facility due to acute on chronic limb ischemia. The patient was started on a heparin infusion, but developed hematuria, which prompted its discontinuation. Given that the patient had undergone a kidney biopsy four days prior to admission, the patient underwent a noncontrast CT scan to rule out a perinephric hematoma in the setting of the new hematuria. Based on the imaging findings in combination with arterial duplex, the patient was sent to the operating room for a right common femoral, profunda femoris, and superficial femoral endarterectomy with Y patch angioplasty. The patient received general anesthesia for the operation, and upon completion, the patient had a return of a palpable right pedal pulse. Intraoperative hemoglobin was 7.0 g/dL, and two units of PRBCs were administered towards the end of the case. One hour after the start of the first transfusion, the patient was noted to have decreasing saturations despite 100% fraction of inspired oxygen (FiO2). He was noted to have ventilator dyssynchrony and high peak pressures. An immediate chest X-ray was obtained postoperatively and is shown in Figure 1 compared to preoperative imaging. Immediate blood work and arterial blood gas (ABG) were also obtained and shown in Table 1 compared to intraoperative laboratory testing.

**Table 1:** Blood work and arterial blood gas: intra-operative versus post-operative values Serial arterial blood gas showing the development of acute respiratory acidosis and hypoxia immediately postoperative. INR: international normalization ratio; PTT: partial thromboplastin time

Laboratory Study	Intra-Operative	Immediately Post-Operative	Reference range
White Blood Cell Count	7.2 x 10(3)/mcL	20.1x 10(3)/mcL	4.8-10.8 x 10(3)/mcL
Hemoglobin	10.1 g/dl, repeat 7.0 g/dl	14.6 g/dl	12.0-16.0 g/dL
Hematocrit	28.7 %	43.1 %	40-50%
Platelets	198 x 10(3)/mcL	252 x 10(3)/mcL	150-450 x 10(3)/mcL
INR	1.14	-	0.8-1.2
PTT	36.3 seconds	-	25-35 seconds
Sodium	132 mmol/L	137 mmol/L	136-145 mmol/L
Potassium	5.0 mmol/L	6.0 mmol/L	3.5-5.1 mmol/L
Chloride	98 mmol/L	110 mmol/L	96-106 mmol/L
Carbon Dioxide	25 mmol/L	24 mmol/L	24- 29 mmol/L
Creatinine	2.9 mg/dL	2.7 mg/dL	0.5-1.2 mg/dL
Blood Urea Nitrogen	46 mg/dL	38 mg/dL	6-23 mg/dL
Glucose	257 mg/dL	136 mg/dL	70-99 mg/dl
Arterial Blood Gas			
pH	7.36	7.20	7.35 - 7.45
PaCO2 (mm Hg)	36	59	35-45 mm Hg
PaO2 (mm Hg)	405	43	75-100 mm Hg
HCO3 (mmol/L)	21	23	22-26 mmol/L
O2 Saturation (%)	100	68	95-100%

**Figure 2:**
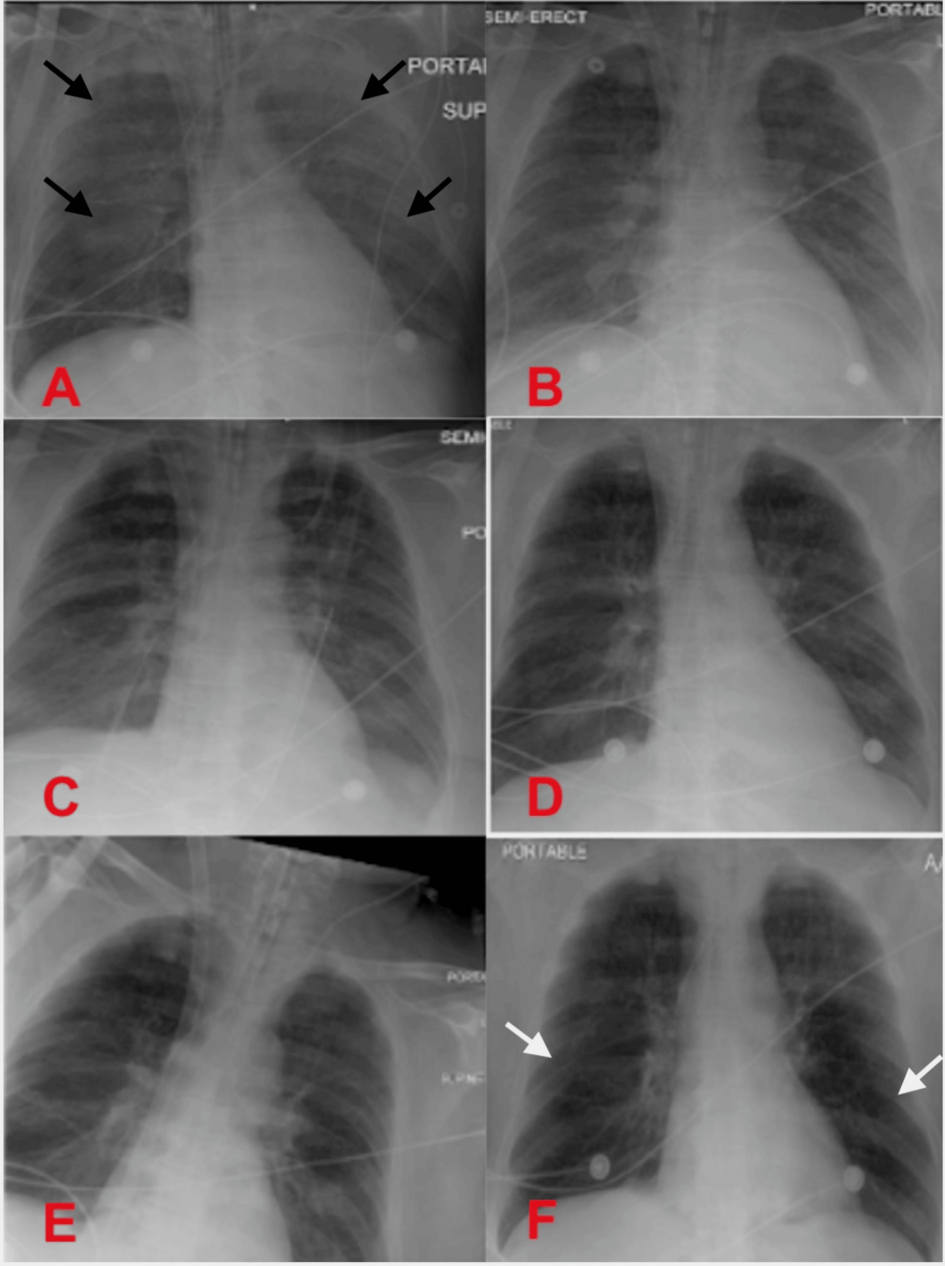
Serial chest X-rays while on ECMO showing interval gradual improvement of initial bilateral infiltrates (black arrows) till complete resolution (white arrows) on Day-7 (A) Day-0, (B) Day-2, (C) Day-3, (D) Day-4, (E) Day-6, and (F) Day-7 at time of decannulation ECMO: extracorporeal membrane oxygenation

Due to laboratory and imaging findings, the patient's positive end expiratory pressure was increased to 24 cm H2O, and the patient was placed on sedation and paralytics. The patient was initiated on epoprostenol, bronchodilators, stress-dose diuretics, and high-dose steroids. Intracardiac shunting and pulmonary embolism were both ruled out via transesophageal echocardiogram (TEE). At this time, the patient was presumed to have TRALI, and given the precipitous decline in his respiratory function despite maximal therapy, the decision was ultimately made to place the patient on VV-ECMO via a bi-caval dual lumen catheter under TEE guidance. He was also initiated on continuous renal replacement therapy (CRRT) due to acute renal failure. Daily serial chest X-rays while on ECMO were obtained and are shown in Figure 2.

Ultimately, the patient's respiratory and renal function significantly improved, and he was decannulated on postop Day-7 and ultimately extubated on postop Day-8. The patient remained stable post-decannulation and was weaned off of CRRT and transferred to acute rehab on postop Day-14.

## Discussion

TRALI, characterized by acute hypoxemic respiratory failure within six hours of blood transfusion and bilateral infiltrates on chest radiography, is a rare but potentially fatal complication of blood transfusion with an incidence of less than 1% per unit transfused [1]. According to the 2017-2021 FDA report on blood collection and transfusion, TRALI is now the second-highest cause of transfusion-associated fatality, with transfusion-associated circulatory overload (TACO) being the leading cause [2]. The present case describes the use of VV-ECMO for the treatment of TRALI in the ICU.

Patients at risk of TRALI often include postoperative or critically ill individuals, with a higher incidence observed in plasma transfusions compared to platelet and red blood cell transfusions [3]. Research using mouse models indicates that patients with low interleukin-10 levels and high C-reactive protein are particularly vulnerable [4,5]. Furthermore, autologous donors who present a significant risk of inducing TRALI in recipients often include those who are female, have a history of multiple pregnancies, and exhibit high levels of anti-human leukocyte antigen class II antibodies (HLA antibodies) and anti-human neutrophil antigen antibodies (anti-HNA antibodies) [6].

The pathophysiology of TRALI involves a "two-hit" mechanism. This begins with neutrophil sequestration in the pulmonary vasculature and neutrophil activation due to a proinflammatory state, corresponding to higher levels of inflammatory markers such as interleukin 8 and CRP. The second hit is the transfusion of blood products admixed with antibodies that activate the recipient's neutrophils to unleash cytokines and reactive oxygen species, culminating in endothelial damage and capillary leak of the lung parenchyma [7-10]. The TRALI classification was updated recently into type I and type II, each with specific clinical criteria [11]. Type I includes an acute onset of hypoxia, bilateral pulmonary edema, and the absence of left atrial hypertension within six hours of transfusion. Conversely, patients grouped into TRALI type II include the clinical features of type I but with a previously stable pulmonary status in the 12 hours preceding transfusion. These patients may also possess risk factors for ARDS, coinciding with the onset of symptoms with transfusion [11].

In the present case, the patient developed hypoxemia shortly after red blood cell transfusion during a vascular procedure. Distinguishing TRALI from TACO, a common differential diagnosis, is crucial for proper diagnosis and management. While TRALI presents with respiratory distress due to an immune-mediated process, patients with TACO typically present with signs and symptoms of fluid overload and may have a history of congestive heart failure [8]. While both TRALI and TACO can present with respiratory symptoms following transfusion, careful assessment of clinical features and underlying mechanisms can differentiate between the two conditions.

Treatment for TRALI includes immediate cessation of transfusion followed by supportive care with high-flow oxygenation. However, approximately 72% of TRALI patients will require ventilatory support [12]. Epoprostenol was used as a supportive respiratory measure prior to ECMO cannulation in this case. Although INO, an alternative pulmonary vasodilator, has been reported to improve oxygenation status in some TRALI cases, its overall impact on mortality or length of stay remains inconclusive [13]. When mechanical ventilation is necessary, ECMO may be considered, as guidelines suggest following ARDS principles of respiratory support [14]. Although the reported utilization of ECMO for the management of TRALI is limited to a few case reports [9,15-17], it appears to be a viable management option. The reports describe patients who were cannulated at various points either intraoperatively or relatively soon afterwards, and they not only survive the lung injury but are often extubated immediately after ECMO decannulation. Despite potential complications, ECMO serves as an important temporary treatment for severe TRALI cases [9].

## Conclusions

VV-ECMO can be used for acute respiratory failure when initial interventions fail. Although TRALI management routinely involves supplemental oxygenation, cessation of transfusion, and other supportive measures, there is limited research on TRALI recovery outcomes with ECMO. Further studies are necessary to show potential patient outcomes with ECMO, including exploring alternative methods such as pulmonary vasodilators.
